# Supplementary material for: Endocrine and Metabolic Impact of Oral Ingestion of a Carob-Pod-Derived Natural-Syrup-Containing D-Pinitol: Potential Use as a Novel Sweetener in Diabetes
Source: Pharmaceutics. 2022 Jul 30;14(8):1594. doi: 10.3390/pharmaceutics14081594 (PMC9416495; doi:10.3390/pharmaceutics14081594)
Supplement: Supplementary file 1 [file pharmaceutics-14-01594-s001.zip › pharmaceutics-1810720-supplementary.pdf]

SUPPLEMENTARY MATERIALS

Endocrine and metabolic impact of oral ingestion of a carob-pod derived natural syrup containing D-Pinitol: potential use as a novel sweetener in diabetes

Juan A. Navarro [1,2], Juan Decara [1], Dina Medina-Vera [1,2,3,4], Ruben Tovar [1,2], Antonio J. Lopez-Gambero [1,4], Juan Suarez [1,6], Francisco Javier Pavón [1,3], Antonia Serrano [1], Marialuisa de Ceglia [1], Carlos Sanjuan [5], Elena Baixeras [7,*], Fernando Rodríguez de Fonseca [1,]

SUPPLEMENTARY TABLE S1.

Primer references for TaqMan® Gene Expression Assays (Applied Biosystems).

Gene description	Assay ID	Nº accession GenBank	Amplicon Length
**Target genes**			
Fbp1	Rn00561189_m1	NM_012558.3	77
G6pc	Rn00689876_m1	NM_013098.2	64
Pc	Rn00562534_m1	NM_012744.2	97
Pck1	Rn01529014_m1	NM_198780.3	87
Pklr	Rn01455286_m1	NM_012624.3	58
Fasn	Rn01463550_m1	NM_017332.1	148
Acox1	Rn01460628_m1	NM_017340.2	63
Acaca	Rn00573474_m1	NM_022193.1	60
Cox4i1	Rn00665001_g1	NM_017202.1	72
Cox4i2	Rn00585003_m1	NM_053472.1	59
Scd1	Rn00594894_g1	NM_139192.2	86
Cpt1a	Rn00580702_m1	NM_031559.2	64
**Reference genes**			
Actb	Rn00667869_m1	NM_031144.3	91

SUPPLEMENTARY FIGURE S1

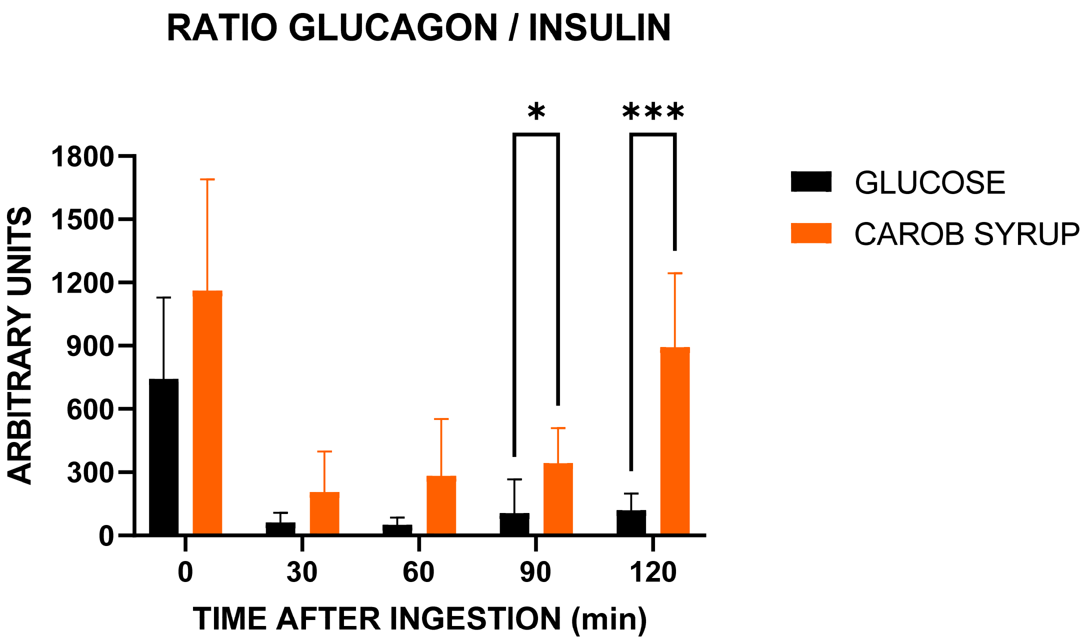

Acute effects on plasma glucagon/insulin ratio in human voluntary subjects of 1) a single oral dose of a glucose solution (50 gr in a 100 ml of water) or 2) a natural carob pod-derived syrup (Innosweet®, 50 gr of carbohydrates in 100 ml water, containing equal amounts of glucose and fructose, and 1600 mg of D-Pinitol). Data are means ± standard error of the mean of 8 subjects for glucose and 9 subjects for carob syrup. (*) $P<0.05$, (***) $P<0.001$ carob syrup *versus* glucose group.

SUPPLEMENTARY FIGURE S2

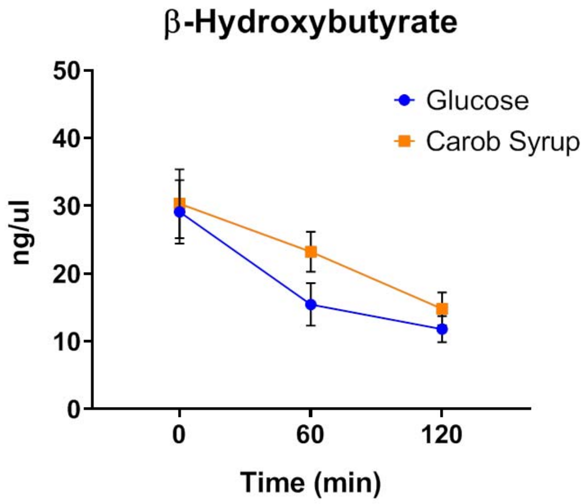

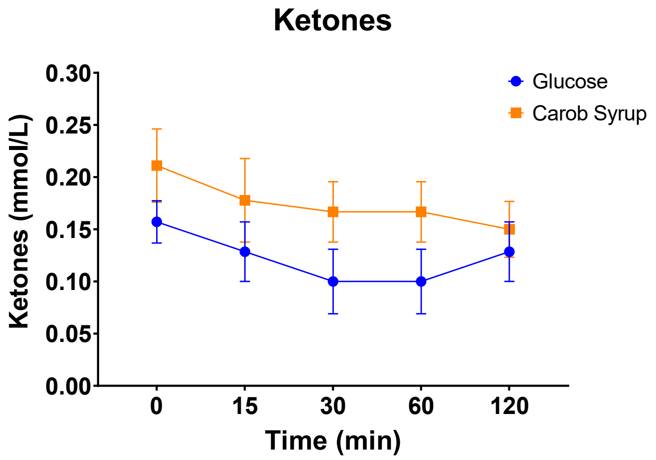

Acute effect on plasma β-hydroxybutirate and plasma ketone levels levels in human voluntary subjects receiving 1) a single oral dose of a glucose solution (50 gr in a 100 ml of water) or 2) a natural carob pod-derived syrup (Innosweet®, 50 gr of carbohydrates in 100 ml water, containing equal amounts of glucose and fructose, and 1600 mg of D-Pinitol). Data are means ± standard error of the mean of 8 subjects for glucose and 9 subjects for carob syrup.

SUPPLEMENTARY FIGURE S3.

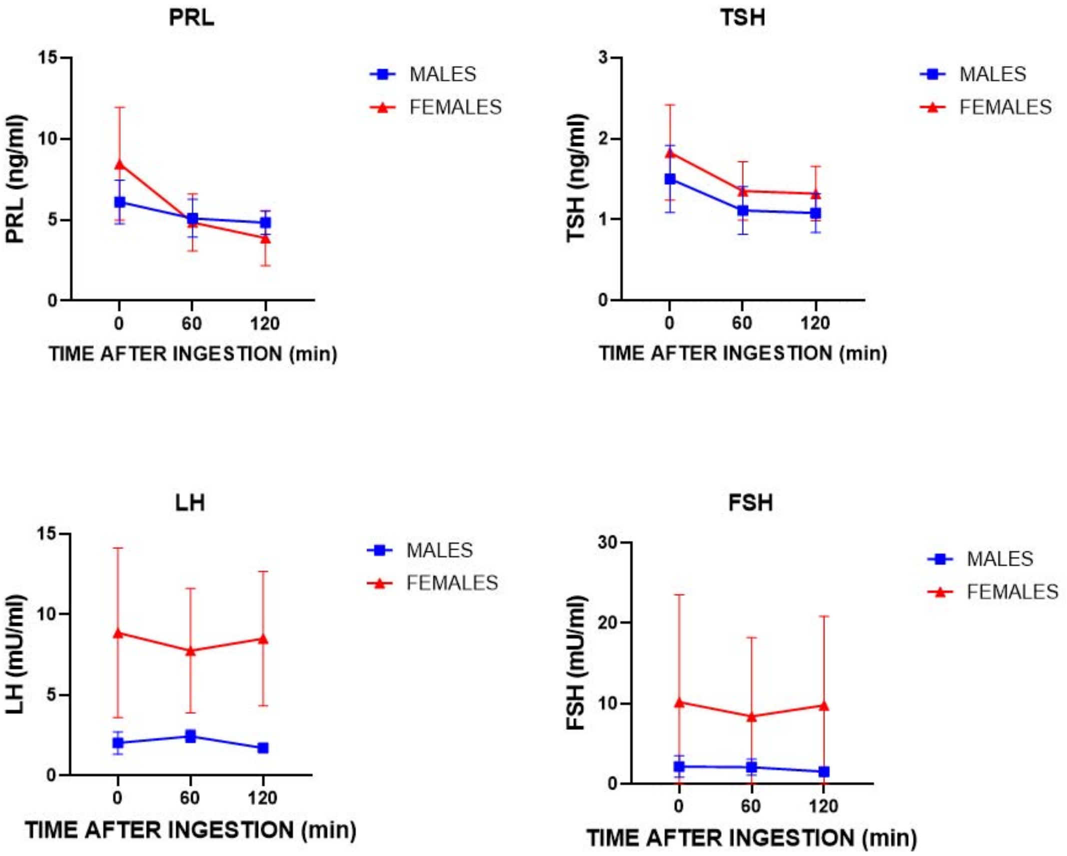

Acute effect in human voluntary subjects of a single oral dose of a natural carob pod-derived syrup (Innosweet®, 50 gr of carbohydrates in 100 ml water, containing equal amounts of glucose and fructose, and 1600 mg of D-Pinitol) on plasma pituitary hormones in male and ramdomly cycling female human subject: Prolactin (PRL), Thyroid-stimulating hormone (TSH), Follicle-stimulating hormone (FSH) and Luteinizing hormone (LH). Data are means ± standard error of the mean of 9 subjects for carob syrup.

SUPPLEMENTARY FIGURE S4

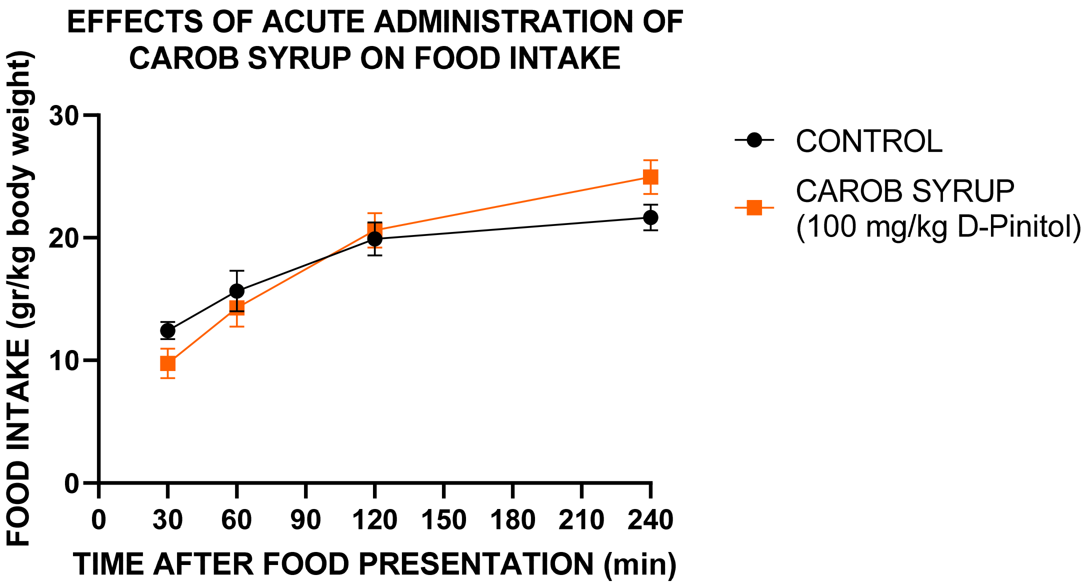

Acute effects a single oral dose of a natural carob pod-derived syrup (Innosweet®, 100 mg/ml, dissolved in 1 ml of water) or 1 ml of water (Control), in animals food deprived for 18 hr. Data are means ± standard error of the mean of cumulative food intaken along a period of 4 hr (8 animals for control group, 9 subjects for carob syrup).

Supplementary Table S2. Plasma and Liver Biochemistry Parameters after 10 days of drinking **water** , or water-diluted **D-Pinitol** (equivalent to 100 mg/kg b.w. /day of D-Pinitol).

	**Water**	**D-Pinitol**
N	10	10
Glucose (mg/dl)	247.0 ± 63.8	269.4 ± 46.3
Creatinin (mg/dl)	0.57 ± 0.38	0.70 ± 0.10
Urea (mg/dl)	21.6 ± 3.2	**41.8 ± 2.8 (*)**
Bilirubin (mg/dl)	0.10 ± 0.09	0.10 ± 0.02
Uric Acid (mg/dl)	1.67 ± 0.29	1.90 ± 0.17
Triglycerides (mg/dl)	146.9 ± 30.1	131.1 ± 9.39
β-Hydroxy butirate (mg/dl)	1005 ± 86	1057 ± 90.8
AST (U/L)	152.6 ± 46.5	254.6 ± 33.18
Insulin (ng/ml))	14.9 ± 1.9	14.5 ± 0.9
Glucagon/Insulin ratio	26.6 ± 4.9	37.3 ± 11.9
Leptin (ng/ml)	14.8 ± 4.6	12.6 ± 3.4
Ghrelin (ng/ml)	0.42 ± 0.13	0.53 ± 0.14
TBARS (Malonyl dialdehyde, μM)	11.1 ± 2.7	8.94 ± 1.09
Total Fat in Liver (mg/g)	40.8 ± 1.3	**30.4 ± 6.9 (*)**
Liver Glycogen (μg/g)	137.7 ± 34.1	107.8 ± 10.6

Data are means ± Standard Deviation. (*) indicates $p<0.05$, ANOVA or Kruskal-Wallis test. AST (Aspartate aminotransferase), TBARS (Tiobarbituric acid reactive species).

SUPPLEMENTARY FIGURE S5.

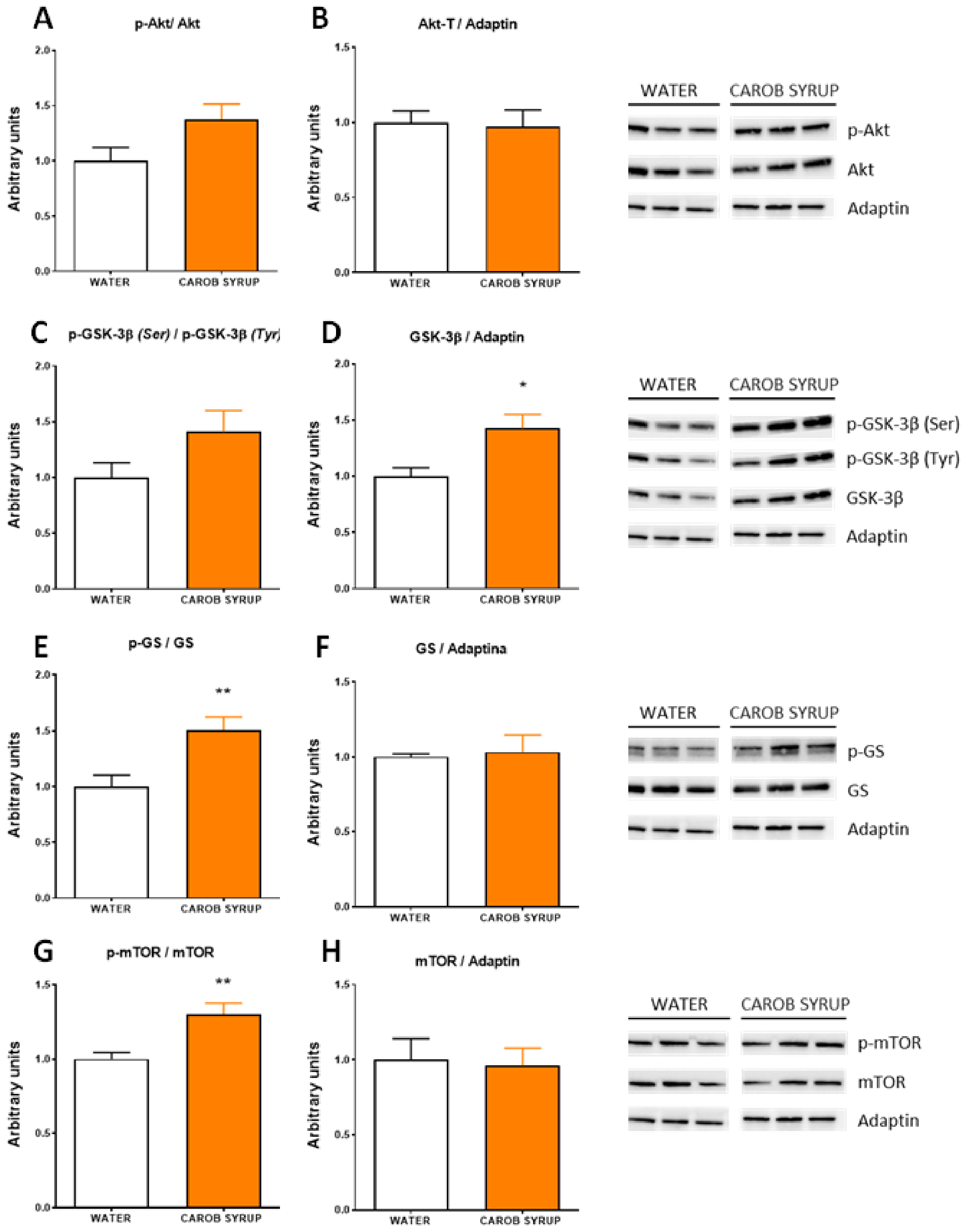

Effects of repeated administration for 10 days of a carob syrup administered in the drinking water on hypothalamus insulin signaling cascade measured by western blot analysis .(**A**) Phospho-protein kinase b/AKT (p-AKT), (**B**) protein kinase b/AKT (AKT), (**C**) Ratio Serine-phosphorylated glycogen synthase kinase 3 β(p-GSK3 β)(Ser) to Tyrosine-phosphorylated glycogen synthase kinase 3 b (p-GSK3 b)(Tyr)), (GSK3 β)(Ser)/ p-GSK3 b)(Tyr)) (**D**) Total GSK3 β, (**E**) Phospho-glycogen synthase (pGS), (**F**) Total Glycogen synthase (GS), (**G**) Phospho-mammalian target of rapamicin (p-mTOR) and (G) Mammalian target of rapamicin (mTOR). Data are means or adaptin-normalized band densities ± standard errors of the mean of 5-8 determinations per group. (*) *P*<0.05, (**) *P*<0.01 carob syrup *versus* water drinking control animals.
